# Small-Seeded Legumes as a Novel Food Source. Variation of Nutritional, Mineral and Phytochemical Profiles in the Chain: Raw Seeds-Sprouted Seeds-Microgreens

**DOI:** 10.3390/molecules24010133

**Published:** 2018-12-31

**Authors:** Bronislava Butkutė, Lukas Taujenis, Eglė Norkevičienė

**Affiliations:** 1 Chemical Research Laboratory, Institute of Agriculture, Lithuanian Research Centre for Agriculture and Forestry, 58344 Kėdainių r., Lithuania; 2 Department of Analytical and Environmental Chemistry, Vilnius University, 01513 Vilnius, Lithuania; lukastaujenis@gmail.com (L.T.); 3 Department of Grass Breeding, Institute of Agriculture, Lithuanian Research Centre for Agriculture and Forestry, 58344 Kėdainių r., Lithuania

**Keywords:** small-seeded legumes, raw seeds-sprouted seeds-microgreens, proximate composition, mineral composition, isoflavones, triterpene saponins, condensed tannins

## Abstract

Growing public concerns about health haves prompted the search for novel food sources. The study is focused on the seeds, sprouted seeds and microgreens of *Trifolium pratense, T. medium*, *Medicago sativa*, *M. lupulina, Onobrychis viciifolia, Astragalus glycyphyllos* and *A. cicer* species as a potential source of value-added food ingredientsr. The samples were analysed for nutritional (wet chemistry, standard methods) and mineral (atomic absorption spectroscopy, UV-Vis spectrophotometry) profiles, isoflavones (ultra-performance liquid with diode array detector –UPLC-DAD), coumestrol (UPLC-DAD), condensed tannins (CT) (vanillin-H_2_SO_4_ assay) and triterpene saponins (UPLC with triple-stage quadrupole MS). In our study, each species displayed high, but species-dependent nutritional, mineral and phytochemical value. All counterparts of legumes were mineral and protein rich. *A. glycyphyllos* samples, especially seeds, were abundant in iron. *Trifolium* spp. were found to be important sources of isoflavones, *Medicago* spp. of coumestrol and saponins, and *O. viciifolia* of CT. The protein and phytochemical contents increased and total carbohydrates decreased from seeds to microgreens.Our findings proved for the first time that seeds, sprouted seeds, and especially microgreens of small-seeded legumes are promising new sources of ingredients for fortification of staple foods with bioactive compounds, minerals and nutrients.

## 1. Introduction

Nowadays, the question is not only to eat for survival, but to be aware of what we eat and know that the food will provide the opportunity to enjoy the quality of life for longer [1]. Natural health benefit products and functional foods embrace a wide range of foods or their ingredients, characterized by diversity of bioactive components which effectively promote health and prevent diseases beyond basic nutrition [2]. Such complex properties are characteristic of legume seeds and their germinated products containing high contents of protein, carbohydrates, minerals and various health promoting phytochemicals.

The *Fabaceae* is one of the largest families of flowering plants, with some 18,000 species classified into 650 genera, which are a significant component of nearly all terrestrial biomes, on all continents (except Antarctica) [3]. Because of their high nutritional value, abundance of minerals and secondary metabolites, grain legumes or pulses have become valuable components of staple and functional foods [4]. The results of archaeological research imply that not only pulses but also some perennial legumes have been consumed as edible or medicinal plants since ancient times. Small legume seeds are commonly recovered from Epipalaeolithic and Neolithic sites in North Africa and South-West Asia, often as a high proportion of the total plant remains [5,6]. Generally, they resembled the seeds belonging to the tribes *Trifolieae* or *Galegaeae* and mostly consisted of *Astragalus* and *Trigonella* species, as well as *Melilotus*, *Trifolium*, *Medicago* spp. and various undetermined morphotypes [5,6]. Their role is uncertain, but it is thought that small-seeded legumes may once have been a human food resource [6].

Traditionally, many herbaceous perennial legumes are used as highly nutritive fodder crops. However, renewed interest in under-utilized plant species for food mainly arises from the finding and promotion of nutritionally relevant attributes [7]. These products can also gain value as functional foods and ingredients. For centuries and in different countries, their young plants, leaves, flowers or seeds have been used as food and in phytotherapy [4,8,9,10,11]. Nowadays, plant material of alfalfa (*Medicago sativa* L.) and red clover (*Trifolium pratense* L.) is sold as bulk powdered herb, capsules, caplets and tablets or seeds in health food stores and online shops. Numerous investigations on the distribution of secondary metabolites in perennial legume species, mostly in *T. pratense* and *M. sativa*, were focused on plant aerial parts [12,13]. More recently, our studies have demonstrated beneficial proximate and phytochemical profiles as well as antioxidant and antimicrobial properties of aerial plant parts of seven temperate perennial species: red clover, zigzag clover (*T. medium* L.), alfalfa, black medic (*M. lupulina* L.), sainfoin (*Onobrychis viciifolia* Scop.), liquorice and cicer milkvetches (*Astragalus glycyphyllos* L., *A. cicer* L.) [14,15,16,17,18]. Much less is known about secondary metabolites in the seeds of perennial legumes. A team of Italian researchers [19] was one of the first to pay attention to flavonoid composition in the seeds of forage legumes, and stated that according to the identified bioactive compounds, they could be of great interest as a potential source of functional compounds for nutraceutical applications. However, much uncertainty still exists about the nutritional and mineral compositions of perennial legume seeds.

The selection of species for the current study was based on the phytochemical composition of aerial plant parts, distribution region, and importance in agriculture or food/phytomedicine. Red clover is widely grown across the world as a forage crop for livestock and poultry and has also been used in folk medicine. Alfalfa is a worldwide important forage crop and has long been used in traditional herbal medicine in China, Iraq, Turkey, India and America for the treatment of a variety of ailments [8]. Even its name alfalfa, by the etymology, coming from the Arabian word “al-fac-facah” which means “father of all foods”, emphasizes the importance of the crop for humans. The two *Astragalus* species chosen for this study, cicer milkvetch and liquorice milkvetch, grow naturally in the temperate climate conditions of Eurasia. The species have been used in traditional medicine as well as a food component in several European countries [9,10]. Sainfoin plants contain condensed tannins (CT) [12,18]. These compounds have attracted interest as antibiotics and antioxidants in human medicine [20]. The decision to include a wild ecotype of zigzag clover in the study was based on the evidence that the concentrations of isoflavones in it were several times as high [15] as those in other legumes (soybean, red clover), which are well known and widely used as sources of phytoestrogens [21]. Black medic is a multifunctional legume that has potential for pasture, green manure, cover cropping, intercropping, and phytoremediation throughout temperate and subtropical regions of the World [22] as well as for medicinal application [23].

Seed sprouting is a widely used natural processing method, which not only improves nutritional properties, decreases levels of antinutritional constituents but also enhances concentrations of bioactive compounds and antioxidant activity resulting in improved nutraceutical properties of seeds and creates a functional component for healthy food production [24,25,26]. Extensive breakdown of seed-storage compounds and synthesis of structural proteins and other cell components take place during germination [27]. Compared with sprouts, microgreens have superior flavour and aroma and present a wider range of textures and colours [28]. Although alfalfa seeds occupy one of the world’s leading positions among the seeds used for health foods, the information on the nutritional, phytochemical and mineral profiles of seeds, sprouted seeds or microgreens is extremely limited and fragmentary [19,26,29,30]. Even less information is available on the chemical composition of red clover seeds and germinated products [19,31]. Moreover, there is a general lack of scientific evidence on this aspect regarding other species selected for the current research. As a result, this study set out to evaluate forage legumes from the new viewpoint as a beneficial source for healthy food through the quantitative determination of the nutritional, mineral and phytochemical compositions in the chain raw seeds-sprouted seeds-microgreens. The study hypothesis was that seeds and sprouts of perennial legumes are of high nutritional value and are rich in various secondary metabolites, which are dependent on plant species and ontogenic stage (raw seeds, sprouted seeds, microgreens).

## 2. Results and Discussion

### 2.1. Proximate Composition

The results in Table 1 show the comparative proximate composition of different legumes in a chain raw seeds-sprouted seeds-microgreens. 

Samples of perennial legumes of all ontogenic stages proved to be valuable sources of protein which amounted to 32.0–36.1 g/100 g in seeds, 37.0–45.2 g/100 g in sprouted seeds and 44.0–58.7 g/100 g in microgreens. In general, seeds and germinated products are characterized by low to relatively high crude fat content (2.85–11.6 g/100 g in seeds, 3.95–9.28 g/100 g in sprouts and 4.13–6.48 g/100 g in microgreens) and moderate levels of ash and carbohydrates (3.97–8.19 and 36.1–46.0 g/100 g in seeds, 4.24–5.35 and 29.7–37.6 g/100 g in sprouts, 5.34–7.31 and 20.0–29.7 g/100 g in microgreens, respectively for ash and carbohydrates).

Among the species, all respective counterparts of *O. viciifolia* (*Ovi*) containedin the highest concentration of protein (35.8 g/100 g of seeds, 45.2 g/100 g of sprouted seeds and 58.7 g/100 g of microgreens) and the lowest concentration of crude fibre (CF) (12.2, 11.4 and 8.7 g/100 g). *M. sativa* (*Msa*) and *M. lupulina* (*Mlu*) samples of all three ontogenic stages had higher fat contents than respective counterparts of other species, except for *T. pratense* (*Tpr*) sprouts in which fat content was of a similar level as in *Msa, Mlu* sprouts. Seeds and sprouted seeds of *A. glycyphyllos* (*Agl*) contained less protein (32.0 g/100 g and 37.0 g/100 g, respectively) and fat (2.85 g/100 g and 3.95 g/100 g, respectively), more CF (16.2 g/100 g and 17.2 g/100 g, respectively) than the same counterparts of the other legumes tested.

The protein content in the counterparts of the studied legume species increased and total carbohydrates (TC) decreased in the following order: seeds-sprouted seeds-microgreens (Table 1). Generally, microgreens contained more ash and CF and less fat than raw and sprouted seeds, except for the concentrations of fat in *Mlu* and fibre in *Ovi* counterparts.

There is a paucity of systematic investigations on chemical composition of perennial legume seeds, sprouts or microgreens. Our results on fat concentration in alfalfa seeds and sprouts are in line with those reported by Márton et al. [29], who have found 10.3 and 9.8% of crude fat on air dry matter basis, in respective counterparts. Dehulled *Ovi* seeds were found to contain 388 g kg^−1^ crude protein (CP) [32]. No other reports were found on the proximate composition of the seeds tested. In general, perennial legume seeds are characterized by higher protein content than most grain legume seeds [33,34,35], except for soybean [33,35], yellow and white lupin [35]. In our study, perennial legumes exhibited higher or similar crude fat and ash and lower TC contents in comparison with grain legumes traditionally used in food [33,34,35], except for soybean and peanut. Results of the current study revealed species-dependent variation in proximate profiles irrespective of the ontogenic stage. This is consistent with previous reports [35] which clearly showed the variation in proximate composition of seeds both between and within legume species.

Similar trends of quantitative changes in nutrients in sprouting seeds of *Fabaceae* plants (pigeon pea, black gram, mung bean, chickpea, cowpea) and pearl millet have been identified by other researchers [36,37,38,39]. Dueñas et al. [34], however, established a significant (*p* < 0.05) decrease of protein contents in sprouted beans and lentils. The lower values of protein in mung bean and soybean sprouts compared to mature grains were observed by Ebert et al. [28]. However, these results were based on the fresh mass basis. When protein concentrations were adjusted to a dry mass basis, it emerged that sprouts of both mung bean and soybean were considerably protein-richer than raw seeds. The increase in protein content during sprouting is attributable to the synthesis of enzyme proteins or a compositional change following the degradation of other constituents [40]. During sprouting, contradictory changes were observed regarding crude fat content. Our results confirmed the existence of species-dependent differences in the crude fat content variation during sprouting. Masood et al. [36] and Devi et al. [41] determined that fat concentration in sprouted seeds of mung-bean, chickpea and cowpea was lower than in raw seeds. According to Bau et al. [40], the decrease in fat content is related to the degradation of nutrients (lipids and carbohydrates) reserve during sprouting whose essential purpose is to provide the energy required for protein synthesis in plant growth. It is also likely that fatty acids are oxidized to carbon dioxide and water, generating energy for germination and biosynthesis of new compounds. On the other hand, the experimental data are rather controversial. Machado et al. [38] and Khalil et al. [39] observed that crude fat content significantly increased in sprouting grain legume seeds. However, Maneemegalai and Nandakumar [37] found that fat and ash contents in sprouted seeds were not altered. The increase in fat may simply reflect the loss of dry matter, mainly in the form of carbohydrates, due to respiration during sprouting [41]. As TC decrease, the percent ratio of other nutrients increases. That is clearly evidenced by our results as well as by the observation of the aforementioned researchers. Dueñas et al. [34] found that germination process produced a significant (*p* < 0.05) increase in ash and TC. The increase in ash in microgreens, observed in the current study, can be associated with translocation of minerals from the seed to the vegetative mass.

### 2.2. Mineral Profile

The variation of mineral content in the chain raw seeds-germinated seeds-microgreens of small-seeded legumes is given in Table 2. 

Significant differences (*p* < 0.05) were noted in mineral composition among the legume species within the samples of the same ontogenic stages (raw seeds, sprouted seeds and microgreens) as well as among the samples of different ontogenic stages within the same legume species. Potassium contents were higher than those of the other minerals in all samples, irrespective of the species and ontogenic stage. *Ovi* accumulated significantly more potassium in seeds, sprouted seeds and microgreens (1.77, 1.67 and 1.98 g/100 g, respectively) than on average (AVG) for all species analysed (1.35, 1.37 and 1.59 g/100 g, respectively). Depending on the legume species and ontogenic stage, P concentration varied from 0.554 g/100 g in raw seeds of *Mlu* to 1.00 g/100 g in *Ovi* microgreens, Ca ranged from 0.238 g/100 g in *Tpr* microgreens to 0.789 g/100 g in raw seeds of Mlu, and Mg ranged from 0.227 g/100 g in sprouted seeds of *Ovi* to 0.437 g/100 g in sprouted seeds of *Tpr*. Legumes differed considerably in the concentration of iron. Among the tested species, in the samples of all ontogenic stages, the significantly lowest Fe concentrations were found in *Mlu* samples (9.20 mg/100 g in seeds, 13.9 mg/100 g in sprouted seeds and 14.8 mg/100 g in microgreens) and the samples of *Agl* were distinguished by extremely high Fe concentrations, which significantly (*p* < 0.05) decreased in the following order: raw seeds (73.4 mg/100 g)-sprouted seeds (34.5 mg/100 g)-microgreens (27.2 mg/100 g). Zinc concentrations in the seeds of *Tpr*, *Ovi* and *A. cicer* (*Aci*) were high, more than 6 mg/100 g, while the concentration of the element in *Mlu* seeds was almost twice lower, 3.35 mg/100 g. Among the species, samples of *Mlu* and *Agl* were characterised by the highest Ca to P ratio in all ontogenic stages, while in clovers it was the lowest. In the chain from seeds to microgreens, the potassium content varied little and the trend of variation was irregular: a visible, but not always significant increase in its amount was observed in the chain for milkvetches, alfalfa and sainfoin. Most of the investigated species were characterized by a significant decrease (*p* < 0.05) in the Ca content in the chain from seed to microgreens, with the exception of medics-the lowest Ca content was determined in their sprouted seeds. Generally, the concentration of phosphorus increased (*p* < 0.05) from raw seeds to microgreens for all the tested species. As a result, Ca to P ratio was considerably lower in microgreens than in raw and sprouted seeds. In microgreens, it was by 27.2% (*Msa*)–58.5% (*Tpr*) and in sprouted seeds by 12.8% (*Aci*)–34.3% (*Mlu*) lower than in raw seeds. The Ca to P ratio close to 1 (0.898–1.424) was observed only in raw seeds of *Msa*, *Mlu*, *Ovi*, *Agl* and *Aci* as well as in sprouted seeds of *Mlu* and *Agl*. During germination, Zn increased several times in all species. In the investigated chain, Fe concentration also increased, although not as fast as Zn, in all species except for the *Astragalus* species. During the entire chain of *Agl* ontogenic stages, a significant decrease (*p* < 0.05) in Fe content was observed; nevertheless it remained very high even in microgreens (27.2 mg/100 g) and much higher compared to Fe content in the other species. Currently, mineral malnutrition is considered to be among the most serious global challenges to humankind and is avoidable [42]. According to the list of priorities of dietary supplement constituents [43], Ca, K, Mg, Zn and Fe are categorised as constituents of the highest priority. The data on mineral quantification in seeds of perennial legume genera are limited. Therefore it is only possible to compare the mineral composition as well as trends of its changes in sprouting seeds of traditional edible fabaceous plants. In regard to mineral composition of raw seeds, our results clearly showed, that small-seeded legumes are generally richer in most elements than important grain legumes, including chickpea, cowpea, lentil, green pea [44], black beans, white beans and pigeon beans [45], mungbean, soybean [33], conventional and non-conventional flours, including flours of soybean, split pea and faba bean [11]. The concentrations of potassium documented in Özcan & Al Juhaimi study [46] were higher than those in perennial legume seeds in our study. A special attention should be paid to the abundance of Fe in the seeds of small-seeded perennial legumes, in particular, in *Agl* (73.4 mg/100 g) and *Aci* (28.6 mg/100 g). It is noteworthy to indicate the superiority of the seeds of perennial legumes (0.676–1.424, Table 2) over the grain legumes (0.39–0.78) [44], soybeans (0.449–0.648) [46] regarding the Ca:P ratio, which should be not less than 1.0 [44]. A low Ca:P ratio in the diet potentially has adverse health effects, including arterial calcification, bone loss, and death [47]. According to Kemi et al. [48], low habitual dietary Ca:P ratios are common in Western diets. The estimated mean dietary Ca:P ratio from foods and beverages for the period 2001–2014 in a nationally representative sample of 34,741 US adults, 20+ years old (NHANES 2001–2014) was 0.689 mg calcium per mg phosphorus intake from foods and beverages [49]. Food supplementation with seeds of *Mlu*, *Ovi* and *Agl* may be one of the ways to optimize the Ca:P ratio in vegan diets.

Generally, the effects of ontogenic stage in the chain raw seeds-sprouted seeds-microgreens on the mineral profile of legume species has not been investigated before. The available data on the distribution of minerals in the seeds of legume species and the trend of their variation between raw seeds and sprouted products is often contradictory. In regard to mineral composition of seeds and sprouted products of perennial legumes, only one publication by Plaza et al. [26] was found. They suggest that the levels of minerals significantly increased in sprouting *Msa* seeds compared to raw seeds. However, higher values of respective mineral concentrations in raw seeds and smaller differences between raw and sprouted seeds for *Msa*, except for Zn concentration were determined in our study. Yet, the authors [26] have indicated that the changes were uneven and depended on the type of seeds and element. For example, *Msa* sprouts contained 3 times more iron than dry seeds, whereas during sprouting of soybeans, Fe concentration decreased, and in wheat grain the changes were not significant. Özcan and Al Juhaimi [46] confirmed this observation by demonstrating that both trend and level of mineral composition variation between untreated and sprouted soybean seeds depended on plant genotype. Sangronis and Machado [45] found that Ca, Zn content was higher, while Fe concentration was lower in sprouted seeds of *Phaseolus vulgaris* and *Cajanus cajan* than that in raw seeds. The trend of change in Mg content depended on the plant species. Devi et al. [41] and Dave et al. [50] observed significant increase in calcium content in cowpea sprouts. There was an insignificant increase in iron content in sprouted seeds of cowpea genotypes [50]. Bains et al. [51] also reported that soaking and different sprouting periods do not give any significant variation in iron content of mung-bean and cowpea while chickpea had a reduction in iron content when soaked and germinated. The authors observed a significant decrease in calcium and zinc levels in soaked and germinated mung-bean and cowpea seeds.

Micronutrient deficiencies are a global problem concerning two billion people [52]. Iron and zinc are still the most widespread deficient micronutrients in global food systems, known as ‘hidden hunger’ especially among children under the age of 5 and women of childbearing age [53,54]. Iron and zinc are also currently of the greatest concern when considering the nutritional value of vegetarian diets [55]. Despite the large differences among the species and ontogenic stages in Fe content: from 9.20 mg/100 g in seeds of *Mlu* to 73.4 mg/100 g in seeds of *Agl*, many investigated counterparts were higher in iron than edible tissues of common crops, except for some genotypes, generally, of leafy vegetables [42] and higher than in staple foods and conventional food sources [54]. The increased Zn concentrations in sprouted seeds and microgreens of perennial legumes make them the potential sources for food fortification with the element. Moreover, sprouting improved the availability of Fe and Ca from faba bean, soybean [56] and Zn and Mg in pea seeds compared with raw pea [57]. Thus, it can be hypothesized that the sprouted seeds and microgreens of perennial legumes are a good source of Fe and Zn with possibly increased bioaccessibility.

### 2.3. Bioactive Compounds

Numerous significant differences (*p* < 0.05) of isoflavone composition among both the species of legumes and ontogenic stage (raw seeds, sprouted seeds or microgreens) were observed (Table 3).

Like young plants [18] the seeds, sprouted seeds and microgreens of clover species accumulated several times more isoflavones (10.8, 60.9 and 141.8 mg/100 g on AVG for clover species, respectively) than the other species tested (limits of quantification <LOQ, 3.56 and 4.4 mg/100 g on AVG for *Medicago, Onobrychis* and *Astragalus* species, respectively). In raw seeds, two isoflavones (formononetin and biochanin A) were quantified only in *T. medium* (*Tme*) (10.7 and 7.9 mg/100 g, respectively) and *Tpr* (2.0 and 1.0 mg/100 g, respectively); only traces of these isoflavones, i.e., <LOQ, were detected in seeds of other plant species. Daidzein and genistein concentrations were <LOQ in all raw seeds of the tested legumes. Quantifiable concentrations of formononetin and biochanin A were found in germinated products of all legume species with the highest concentrations in microgreens (from 2.1 and 1.2 mg/100 g, respectively, in *Aci* microgreens to 117.5 and 33.4 mg/100 g, respectively in *Tme* microgreens). In most cases the microgreens contained the highest overall concentration of isoflavones, followed by the sprouted seeds and raw seeds. However, the differences between sprouted seeds and microgreens were insignificant for formononetin concentration in *Mlu* and *Ovi* as well as for biochanin A concentration in *Msa*, *Mlu*, *Ovi* and *Aci*. There were only traces (below the LOQ value) of daidzein and genistein in the extracts of all the samples of *Msa, Mlu, Ovi* and *Agl, Aci.*

Like in the above ground part of the legume plants [16,18], in seeds and germinated products, saponins and coumestrol were specific to *Msa, Mlu* and CT to *Ovi*, therefore, Table 4 shows the change in these compounds only in the samples of the aforementioned species. Like isoflavone concentrations, the contents of other phenolics compounds (CT and coumestrol) and non-phenolic metabolites as triterpene saponins (TS) increased in the following order: raw seeds < sprouted seeds < microgreens. Samples of *Mlu* were significantly richer in coumestrol (*p* < 0.05) than the respective samples of *Msa*, while *Msa* seeds and microgreens contained significantly more (*p* < 0.05) TS than those of *Mlu*. Saponin content in sprouted seeds of *Msa* and *Mlu* differed insignificantly.

There is a paucity of data on the quantification of isoflavones and other compounds in the chain raw seeds-sprouted seeds-microgreens of legume species tested in our work. The information on the contents of phytoestrogens in foodstuffs collected by Forslund and Andersson [58] for phytoestrogen distribution in foods of Nordic markets shows that *Msa* sprouts contained much less isoflavones (0.0152 mg/100 g) and clover seeds had similar content (2.152 mg/100 g) to that found in our study. Meanwhile researchers of South Europe [59] have documented higher contents of phytoestrogens in *Msa* sprouts. Budryn et al. [31] reported that germination causes a rapid increase in the content of isoflavones in *Tpr* seeds. Furthermore, the authors showed that prolongation of sprouting led to an increase in free aglycones content and the predominant isoflavone of the *Tpr* sprouts was formononetin, which is in agreement with our findings. More comprehensive investigations on the change in phytochemical composition during sprouting were carried out with other legume species, mainly pulses. The studies indicate that the main isoflavones in chickpea and other pulses are substantially increased during the seed germination [60]. In raw beans, only one genistein derivative was detected in low concentration (0.127 mg/100 g), whereas in germinated beans the total isoflavones increased by up to 2.99 mg/100 g [61]. Particularly in chickpea seeds, the total isoflavone contents were by over 100-fold higher, mainly due to the increase in formononetin and biochanin A level; however, soybean germination had lesser impact on the change of isoflavones content which increased by 43.6% [60]. Lee et al. [62] observed that the total isoflavone concentration increased from 80.4 mg/100 g in soybean seeds to 191.2 and 217.4 mg/100 g in green and yellow soybean sprouts, respectively. The increase in isoflavone content in germinated soy samples was also reported by Ebert et al. [33], Lin and Lai [63] and Plaza et al. [26]. Lin and Lai [63] indicated that the contents and compositions of isoflavones and other bioactive compounds varied greatly between the species of legumes and their cultivars due to genetic characteristics. Jeon et al. [64] claim that isoflavone levels decrease in germinating soybeans; however, an increment in coumestrol concentration from raw to germinated soybeans was observed.

There is no consensus on the germination impact on the concentrations of saponins and CT in various conventional and non-conventional legumes. Our results on saponin compounds and their increase in *Msa* seeds and sprouts agree with those reported by Oleszek [30]. Shimoyamada and Okubo [65] also observed an increase in saponin contents both in cotyledons and sprouts of soybean during germination, especially when using light irradiation. However, in the review paper Singh et al. [66] have indicated that soaking followed by sprouting diminishes the saponin level in pulses by 9–66%. Such contradictions can be associated with several factors. Pecetti et al. [67] found that genotype is a key factor for the saponin content, yet the role of temperature, radiation is also important. 

Like for *Ovi* in our study, Aguilera et al. [24] found that total proanthocyanidins significantly increased in germinated mucuna (50%) and cowpea (40%). Similar levels were found in dolichos. The extension of the germination time caused an increase in proanthocyanidin level in mung-bean [68]. Sangronis and Machado [45], however, observed 14.3% reduction in tannins in germinated pigeon beans, 19% in black beans and 36.2% in white beans. Germination process also reduced tannins in *Vigna unguiculata* (L.) Walp. subsp. *unguiculata* [69]. Świeca et al. [70] reported a reduction in the content of CT during soaking and germination of lentil seeds; however, it should be noted that the authors discussed the data on a fresh mass basis. Concentrations expressed on a fresh mass basis may be influenced by changes in moisture content presenting a dilution effect on the concentration of phenolics [25] and other components. Moisture content in sprouted seeds is several times higher than that in raw seeds [26] and dry matter of microgreens makes up approximately 5% of fresh mass [28].

Compared with sprouts, microgreens are superior not only according to sensory properties [28] but, as our findings show, they are also richer in phytochemical composition. However, no scientific data are currently available on the comparative fluctuation of bioactive compound contents among raw seeds, sprouted seeds and microgreens or at least between sprouted seeds and microgreens in legumes. Ebert et al. [28] found that microgreens of amaranth had higher content of α-carotene, β-carotene, violaxanthin, lutein, and neoxanthin compared to sprouts.

In summary, our studies on small-seeded legumes corroborated the findings of Ebert et al. [28] that germinated seeds, particularly microgreens, have more bioactive compounds than raw seeds. Budryn et al. [31] stated that *Tpr* sprouts can be considered as a source of phytoestrogens with high biological activity and as a dietary supplement reducing menopausal symptoms. Sprouted seed and sprouts are vegetables that can grow in any climate and season. Germination is a simple technological process of short growth cycle, easy to apply, has minimal cost and offers year-round constant, fresh and nutrient-dense produce [28]. According to Klopsch et al. [71] legume-derived components, including seeds and microgreens, could serve as natural ingredients for enhancing health-promoting secondary plant metabolites in wheat products. Thus, our findings revealed that seeds and germinated products of small-seeded legumes are promising novel sources of healthy food and have the potential as ingredients for fortification of staple food with bioactive compounds, minerals and nutrients. In the present work, quantified secondary metabolites (phytoestrogens, triterpene saponins, condensed tannins) possess therapeutic benefits that have not only been used in veterinary and folk medicine, but have also attracted the interest of official medicine [72]. These compounds have been related to antihypercholesterolemia, antidiabetic, antimenopause, anti-inflammatory, anticancer, anthelmintic, cardioprotective and other healthy beneficial effects.

## 3. Materials and Methods

### 3.1. Materials

The collection of seeds of perennial legumes included seven species from four genera and involved four Lithuanian commercial cultivars and three wild ecotypes (Table 5).

The seeds were multiplied in the germplasm collection, established in a field trial in 2012 in the Central Lowland of Lithuania (55°23′49″ N; 23°51′40″ E), at Institute of Agriculture, Lithuanian Research Centre for Agriculture and Forestry. The soil of the experimental site was *Endocalcari-Epihypogleyic Cambisol* with pH 6.8, 18.4 g/kg organic carbon, 50.2% sand, 29.6% silt, and 20.2% clay in 30 cm topsoil layer. No herbicides and fertilizers were applied in the collection nursery.

### 3.2. Seed Sprouting

Prior to germination, impurities and broken seeds were carefully removed and *Ovi* were de-hulled. Seeds of each species were well rinsed with tap water, then with sterile distilled water and placed in the 1000 mL glass sprouting jars (Eschenfelder GmbH, Hauenstein, Germany) with stainless steel mesh tops for easy rinsing. The seeds were soaked in sterile distilled water for 3–12 h depending on the size at ambient temperature until they had absorbed the amount of water sufficient for germination. The soaked seeds were rinsed several times with distilled water. Water was drained prior to sprouting. The jars were placed on a stainless steel rack to hold jars inverted at an angle for drainage and the rack with jars was put on the ceramic drip trays. Seeds were germinated at room temperature (23–24 °C) under natural light until the sprouts attained 1.5–3 cm. During sprouting, seeds were rinsed with sterile distilled water and drained every day.

### 3.3. Growing of Microgreens

Microgreens were grown without soil in Eschenfelder sprouting boxes and Eschenfelder sprouting dishes with sieve K for sprouts of small seeds. The washed, soaked and rinsed seeds (Section 2.2) were evenly spread on the sterilised steel sieves of sprouting boxes or dishes. The germinating seeds, and later seedlings, were moistened 2–3 times per day by spraying distilled water. After the first true leaves had emerged, microgreens were picked from seeds manually.

### 3.4. Sample Preparation

Samples of sprouted seeds and microgreens were freeze-dried. Sublimation/lyophilisation was performed in a Sublimator 3 × 4 × 5 (ZIRBUS Technology GmbH, Bad Grund (Harz), Germany), the condenser temperature was −85 °C, and the vacuum was 2 × 10^−6^ mPa, the samples were frozen at −40 °C in a laboratory freezer, and then left in the freeze-drier for 72 h. Both seeds and freeze-dried samples were ground to pass a 1 mm screen. Three samples per replication were prepared.

### 3.5. Proximate Analysis

Samples of raw seeds, sprouted seeds and microgreens were analysed for protein, fat, CF, ash, and digestible (total) carbohydrate contents according to the methods described by Owusu-Apenten [73]. Crude protein (CP) was determined by the Kjeldahl method with a conversion factor of 6.25; crude fat was measured gravimetrically by the continuous Soxhlet extraction with hexane. Crude fibre (CF) was estimated by successive acid and alkaline hydrolyses of insoluble residues. Crude ash (ash) content was determined as the mass left after full sample incineration at (550 ± 10) °C. Total carbohydrate (TC) content was estimated by difference remaining after subtracting the contents of CF, CP, fat and ash. Data of proximate analysis were expressed in g of nutritional component per 100 g on a dry matter basis (g/100 g).

### 3.6. Determination of Minerals

Essential minerals, such as potassium (K), calcium (Ca), magnesium (Mg), iron (Fe), zinc (Zn) were quantified by flame atomic absorption spectroscopy (AAS) using a model AAnalyst 200 system (Perkin Elmer, Waltham, MA, USA) after wet digestion with the nitric acid and hydrogen peroxide [74]. Parameters of the AAS instrument were chosen in accordance with the manufacturer’s instructions. Total phosphorous (P) was determined after sulfuric acid digestion of the samples and reaction with molybdate-vanadate. The absorbance was measured by UV-V spectrophotometer (Cary 50, Varian, Walnut Creek, CA, USA) at 430 nm. Mineral content was expressed as mg of element per 100 g on a dry matter basis (mg/100 g).

### 3.7. Secondary Metabolite Analyses

#### 3.7.1. Reagents

Daidzein (PubChem CID: 52817087, purity ≥ 98%), genistein (PubChem CID: 5280961, purity ≥ 98%), and their 4′-methylated derivatives, formononetin (PubChem CID: 5280378, purity ≥ 99%) and biochanin A (PubChem CID: 5280373, purity ≥ 98%), coumestrol (PubChem CID: 5281707, purity ≥ 95%), vanillin (PubChem CID: 1183, purity ≥ 97%), oleanolic acid (PubChem CID: 10494, purity ≥ 97%) and (+)-catechin hydrate (PubChem CID: 107957, purity ≥ 98%) were purchased from Sigma-Aldrich (St. Louis, MO, USA). LC-MS grade methanol and LC-MS grade acetic acid were obtained from Fluka (Sigma-Aldrich), water was prepared internally, on Milli-Q water purification system (Millipore, Bedford, MA, USA). All other reagents conformed to the specifications defined by the Committee on Analytical Reagents of the American Chemical Society (ACS purity) and were purchased from Sigma-Aldrich.

#### 3.7.2. Reference Standard Solutions

The coumestrol and isoflavones standard stock solutions of daidzein, formononetin, genistein, biochanin A were prepared at concentration level of 250 mg/L in aqueous methanol (1:1, *v*/*v*) and stored refrigerated at 4 °C protected from light. The working standard solutions were prepared daily by dilution of the standard stock solution in the aqueous methanol (1:1. *v*/*v*). The standard solution of (+)-catechin in methanol was prepared at concentration level of 1 g/L and stored at 4 °C protected from light.

#### 3.7.3. Hydrolysis and Extraction of Phytoestrogens

Hydrolysis and extraction of phytoestrogens from the plants and their individual parts was performed simultaneusly. The representative amount of 250 mg sample was hydrolysed at 80–85° for 1.5 h in 10 mL of 2 M HCl methanol/water solution, followed by extraction in the sonic bath for 30 min in the room temperature. The extracts were filtered through 0.2 µm nylon syringe filter and brought to UPLC analysis of isoflavones.

#### 3.7.4. Extraction of Condensed Tannins

Condensed tannins were extracted from 500 mg plant sample with 5 mL of acetone/water containing 0.5% (*m*/*v*) ascorbic acid. Ascorbic acid was necessarily to prevent tannins oxidation during 1-h extraction procedure on vortex. Sample extracts were centrifuged at 3000 × *g* for 15 min and the supernatant was filtered through 0.2 µm nylon syringe filter. Finally, 3 mL of the hexane was added to 1 mL of fine sample solution for chlorophyll extraction. Aqueous layer was separated and taken for CT spectrophotometric analysis.

#### 3.7.5. Hydrolysis and Extraction of Triterpene Saponins

The slightly modified TS hydrolysis and extraction procedure was adopted from Pecetti et al. [75]. 100 µg of the plant sample was treated under reflux for 8 h in 10 mL 2 M HCl aqueous methanol (1:1, *v*/*v*) solution. Methanol was removed under vacuum and the aglycones were extracted with 5 mL ethyl acetate twice. Organic phase was evaporated to dryness, reconstituted with 5 mL of methanol and filtered through 0.2 µm nylon syringe filter prior LC-MS analysis.

#### 3.7.6. Quantification of Phytoestrogens

Free aglycones of phytoestrogens were analyzed on an Acquity UPLC system equipped with an Acquity DAD detector (Waters, Milford, MA, USA) for quantitation and connected to tandem quadrupole time of flight mass spectrometer MicrOTOF QII (Bruker, Hanau, Germany) for identification. Data were collected and managed with HyStar 3.2 (Bruker). Separation was performed on Acquity UPLC BEH C18 (100 mm × 2.1 mm I.D., 1.7 µm, Waters) column with 15 min linear elution gradient according to our published protocol [76]. The coumestrol and four isoflavones: daidzein, genistein, formononetin, biochanin A were quantified by external calibration and the results were expressed in mg per 1 mg of the dry matter. The seven-point linear calibration ranged from 50 mg/L to 100 mg/L for coumestrol and isoflavones respectively. The limits of quantification were defined as the concentration resulting in a signal of ten times the noise level and ranged from 6 µg/g to 10 µg/g [17].

#### 3.7.7. Quantification of Condensed Tannins

CT were quantified by the spectrophotometric vanillin-sulfuric acid assay using external calibration. An aliquot of 10 µL of aqueous CT sample extracts was diluted with pure methanol by the factor of 100 and incubated for 5 min with 2 mL 1.8 M sulfuric acid in methanol and 2 mL of 10 g/L vanillin solution in methanol. The absorbance was measured at 500 nm with UV-Vis spectrophotometer T60 (Oasis Scientific Inc., Taylors, SC, USA). (+)-Catechin was used for calibration and the CT concentration was expressed as mg/g of catechin equivalents (CE) of a dry mass mg(CE)/g. The limit of quantification was estimated from regression curve as described in ICH Q2(R1) and was 3.2 mg(CE)/g [17].

#### 3.7.8. Quantification of Total Triterpene Saponins

Saponins aglycones were analysed on a 1290 Infinity UPLC system connected to tandem mass spectrometer 6410 Triple Quadrupole (Agilent Technologies, Santa Clara, CA, USA). The atmospheric pressure chemical ionization source was set to negative ionization mode and mass analyser was optimized for single ion monitoring. Data acquisition and processing were performed with MassHunter (Agilent Technologies). Separation was performed on an Acquity UPLC BEH C18 column (100 mm × 2.1 mm I.D., 1.7 µm, Waters) with linear elution gradient as described in [17]. The total amount of saponin aglycones was measured using internal calibration with oleanolic acid and the determined concentration was adjusted to dry plant mass. The limit of quantification for the oleanolic acid was estimated form regression parameters and was 0.25 mg/g.

### 3.8. Statistical Analysis

In order to adequately and appropriately compare the proximate, mineral and phytochemical compositions in the chain raw seeds-sprouted seeds-microgreens, which differ in moisture content, all the results are provided on a dry matter basis. The data were subjected to one-way analysis of variance (ANOVA) to determine significant differences in the respective proximate, mineral and phytochemical concentrations (1) among the legume accessions within the same ontogenic stage and (2) among raw seeds, sprouted seeds and microgreens within the same legume species. Analyses of the data were performed with SAS Enterprise Guide 7.1 (SAS Institute Inc., Cary, NC, USA) followed by Duncan’s multiple range test; *p*-values < 0.05 were considered significant.

## 4. Conclusions

The study, for the first time, provides valuable information about the raw seeds, sprouted seeds and microgreens of perennial legume species as a source of health-promoting phytochemicals. *A. glycyphyllos* samples, especially seeds, were abundant in iron. *Trifolium* spp. were found to be important sources of isoflavones, *Medicago* spp. of coumestrol and saponins, and *O. viciifolia* of condensed tannins. The concentration of proteins, fibre and bioactive compounds as well as bioactivity increased in the sequence: raw seeds-sprouted seeds-microgreens, while the content of total carbohydrates decreased. In this chain, the regularities of fat content and mineral composition variation were plant species and component-depended. Overall, our research proved that sprouted seeds, especially microgreens, contain higher concentrations of all the bioactive compounds tested compared with raw seeds.

## Figures and Tables

**Table 1:** The proximate composition (g/100 g ± SD) of raw seeds, sprouted seeds and microgreens of perennial legumes.

Sample	Ash	Crude Protein	Crude Fat	Crude Fibre	Total Carbohydrates
**Raw seeds**					
*Tpr*	4.71 ± 0.34 cdB	34.5 ± 0.19 abC	8.98 ± 0.01 bA	13.8 ± 0.71 abC	38.0
*Tme*	7.32 ± 0.24 bA	34.8 ± 0.22 abB	n.a.	n.a.	n.a.
*Msa*	4.32 ± 0.02 dB	34.7 ± 0.41 abB	11.6 ± 0.07 aA	13.3 ± 0.23 bB	36.1
*Mlu*	3.97 ± 0.03 eC	34.1 ± 0.66 abC	3.10 ± 0.35 dC	12.8 ± 0.77 bAB	46.0
*Ovi*	4.46 ± 0.08 dC	35.8 ± 0.43 aC	6.62 ± 0.21 cA	12.2 ± 0.60 bcA	40.9
*Agl*	8.19 ± 0.10 aA	32.0 ± 0.37 bB	2.85 ± 0.14 dB	16.2 ± 1.00 aA	40.8
*Aci*	5.24 ± 0.23 cA	36.1 ± 0.13 aB	3.78 ± 0.14 dA	14.9 ± 0.69 aA	40.0
AVG*	5.46	34.6	6.15	13.9	40.3
AVG**	4.37	34.8	7.56	13.0	40.3
**Sprouted seeds**					
*Tpr*	4.42 ± 0.37 bB	38.9 ± 0.09 dB	9.28 ± 0.01 aA	15.6 ± 0.88 bB	31.8
*Tme*	4.99 ± 0.01 abB	39.8 ± 0.30 cdA	6.62 ± 0.21 b	15.3 ± 0.61 b	33.3
*Msa*	4.65 ± 0.25 bB	42.8 ± 0.38 bA	8.88 ± 0.33 aB	14.0 ± 0.45 bA	29.7
*Mlu*	4.38 ± 0.31 bB	42.6 ± 0.13 bB	5.14 ± 0.24 cA	13.9 ± 0.67 bA	34.0
*Ovi*	5.35 ± 0.08 aB	45.2 ± 0.41 aB	5.58 ± 0.03 cB	11.4 ± 0.54 cA	32.5
*Agl*	4.24 ± 0.21 bB	37.0 ± 0.26 eA	3.95 ± 0.11 dA	17.2 ± 0.43 aA	37.6
*Aci*	5.29 ± 0.44 aA	40.3 ± 0.03 cA	4.08 ± 0.08 dA	15.0 ± 0.58 bA	35.3
AVG*	4.76	40.9	6.22	14.6	33.5
AVG**	4.7	42.4	7.22	13.7	32
**Microgreens**					
*Tpr*	5.82 ± 0.25 bA	45.7 ± 0.43 bcA	4.84 ± 0.23 bcB	17.5 ± 1.06 aA	26.1
*Tme*	na	na	na	na	na
*Msa*	5.34 ± 0.28 bcA	44.0 ± 0.93 cA	6.48 ± 0.21 aC	14.5 ± 0.32 bA	29.7
*Mlu*	6.03 ± 0.18 bA	48.0 ± 0.14 bA	4.13 ± 0.24 cB	14.4 ± 0.69 bA	27.4
*Ovi*	7.31 ± 0.40 aA	58.7 ± 1.04 aA	5.26 ± 0.16 bB	8.7 ± 0.47 cB	20.0
*Agl*	na	na	na	na	na
*Aci*	na	na	na	na	na
AVG**	6.13	49.9	5.18	13.8	25.8

AVG*: average for all species analysed; AVG**: *Tme*, *Agl* and *Aci* were excluded from average computing; na: not analysed. The different lowercase letters (a, b, c) in the column indicate significant differences (*p* < 0.05) in the respective mineral concentrations among the legume accessions within the identical sample group and the different uppercase letters (A, B, C) in the column indicate significant differences (*p* < 0.05) in the respective mineral concentrations among seeds, sprouted seeds and microgreens within the identical legume accession. Abreviations: *Tpr: T. pratense, Tme: T. medium, Msa: M. sativa, Mlu: M. lupulina, Ovi: O. viciifolia, Agl: A. glycyphyllos, Aci: A. cicer*.

**Table 2:** The variation of mineral composition in the chain raw seeds-sprouted seeds-microgreens of perennial legumes (mean ± SD).

Sample	K	Ca	Mg	P	Ca:P (% from Ca:P in Seeds)	Fe	Zn
	g/100 g					mg/100 g	
**Raw seeds**							
*Tpr*	1.35 ± 0.01 bA	0.491 ± 0.017 cA	0.403 ± 0.015 aB	0.702 ± 0.015 abB	0.699	17.8 ± 0.013 cA	7.11 ± 0.767 aC
*Tme*	1.10 ± 0.09 cB	0.495 ± 0.053 cA	0.309 ± 0.013 bB	0.732 ± 0.070 aA	0.676	10.2 ± 0.002 dB	5.74 ± 0.611 bB
*Msa*	1.27 ± 0.03 bB	0.716 ± 0.089 abA	0.304 ± 0.001 bC	0.749 ± 0.038 aC	0.956	10.5 ± 0.603 dB	5.71 ± 0.450 bC
*Mlu*	1.29 ± 0.01 bA	0.789 ± 0.053 aA	0.289 ± 0.013 cB	0.554 ± 0.010 cC	1.424	9.20 ± 0.310 dB	3.35 ± 0.349 cB
*Ovi*	1.77 ± 0.26 aAB	0.716 ± 0.053 abA	0.243 ± 0.025 dB	0.673 ± 0.003 bC	1.064	10.5 ± 0.811 dC	6.32 ± 0.590 abB
*Agl*	1.32 ± 0.17 bB	0.703 ± 0.053 bA	0.288 ± 0.009 cB	0.592 ± 0.017 cA	1.188	73.4 ± 1.57 aA	5.01 ± 0.979 bcC
*Aci*	1.37 ± 0.05 bC	0.669 ± 0.053 bA	0.254 ± 0.013 dB	0.745 ± 0.029 aA	0.898	28.6 ± 1.04 bA	6.08 ± 0.527 bC
AVG*	1.35	0.654	0.299	0.678	0.986	25.00	5.62
AVG**	1.42	0.678	0.310	0.670	1.036	12.00	5.62
**Sprouted seeds**							
*Tpr*	1.00 ± 0.00 dB	0.352 ± 0.004 cB	0.437 ± 0.059 aA	0.689 ± 0.018 cB	0.511 (−27.0)	16.4 ± 1.435 cA	20.1 ± 0.401 aB
*Tme*	1.52 ± 0.07 abA	0.330 ± 0.004 cB	0.374 ± 0.014 bA	0.729 ± 0.007 bcA	0.453 (−33.1)	21.3 ± 0.334 bA	20.3 ± 1.68 aA
*Msa*	1.31 ± 0.04 cB	0.555 ± 0.004 bB	0.319 ± 0.035 bcB	0.858 ± 0.004 aB	0.647 (−32.3)	16.6 ± 0.482 cA	11.8 ± 0.768 cB
*Mlu*	1.22 ± 0.04 cdAB	0.671 ± 0.005 aB	0.307 ± 0.016 cA	0.717 ± 0.012 cB	0.936 (−34.3)	13.9 ± 0.598 dA	16.2 ± 1.35 bA
*Ovi*	1.67 ± 0.18 aB	0.631 ± 0.003 aB	0.227 ± 0.031 eB	0.790 ± 0.014 bB	0.799 (−24.9)	13.4 ± 0.314 dB	13.6 ± 1.02 bcA
*Agl*	1.40 ± 0.06 bB	0.580 ± 0.001 bB	0.284 ± 0.018 dB	0.603 ± 0.006 dA	0.962 (−19.0)	34.5 ± 1.02 aB	11.4 ± 0.810 cB
*Aci*	1.48 ± 0.02 bB	0.584 ± 0.023 bB	0.246 ± 0.010 deB	0.746 ± 0.014 bcA	0.783 (−12.8)	17.1 ± 0.60 cC	9.50 ± 0.924 cB
AVG*	1.37	0.529	0.313	0.733	0.727 (−26.3)	18.65	14.70
AVG**	1.30	0.552	0.323	0.764	0.723 (−28.6)	15.08	15.43
**Microgreens**							
*Tpr*	1.34 ± 0.06 cdA	0.238 ± 0.012 eC	0.358 ± 0.016 bC	0.819 ± 0.008 cA	0.291 (−58.5)	16.5 ± 0.434 bcA	29.9 ± 1.50 aA
*Tme*	na	na	na	na		na	na
*Msa*	1.49 ± 0.01 cA	0.611 ± 0.019 bAB	0.373 ± 0.068 aA	0.878 ± 0.006 bA	0.696 (−27.2)	17.8 ± 0.902 bcA	27.3 ± 1.69 aA
*Mlu*	1.13 ± 0.04 dB	0.702 ± 0.024 aB	0.293 ± 0.004 cAB	0.852 ± 0.001 bA	0.824 (−42.1)	14.8 ± 1.13 cA	17.9 ± 0.921 bA
*Ovi*	1.98 ± 0.01 aA	0.585 ± 0.038 bC	0.299 ± 0.001 cA	1.001 ± 0.009 aA	0.584 (−45.1)	19.8 ± 0.511 bA	13.3 ± 1.24 cA
*Agl*	1.79 ± 0.03 bA	0.446 ± 0.038 cC	0.340 ± 0.022 bA	na		27.2 ± 0.733 aC	19.6 ± 1.45 bA
*Aci*	1.81 ± 0.03 bA	0.339 ± 0.038 dC	0.326 ± 0.022 bcA	na		21.5 ± 0.697 bB	30.0 ± 1.57 aA
AVG*	1.59	0.487	0.332	0.888	0.599 (−60.6)	19.60	23.00
AVG**	1.49	0.534	0.331	0.888	0.599 (−40.8)	17.23	22.10

AVG*-average for all species analysed; AVG**-Tme, Agl and Aci were excluded from average computing; na-not analysed. The different lowercase letters (a, b, c) in the column indicate significant differences (*p* < 0.05) in the respective mineral concentrations among the legume accessions within the identical sample group and the different uppercase letters (A, B, C) in the column indicate significant differences (*p* < 0.05) in the respective mineral concentrations among seeds, sprouted seeds and microgreens within the identical legume accession. *Abbreviations: Tpr-T. pratense, Tme-T. medium, Msa-M*. sativa, *Mlu*-*M. lupulina*, *Ovi*-*O. viciifolia*, *Agl*-*A. glycyphyllos*, *Aci*-*A. cicer*.

**Table 3:** The alteration in isoflavone concentrations (mg/100 g ± SD) in the chain raw seeds-sprouted seeds-microgreens of perennial legumes.

Sample	Formononetin	Biochanin A	Daidzein	Genistein	Sum of Isoflavones
**Raw seeds**					
*Tpr*	2.0 ± 3.3 bC	1.0 ± 0.1 bC	˂LOQ nc C	˂LOQ nc C	3.0
*Tme*	10.7 ± 0.8 aC	7.9 ± 0.8 aC	˂LOQ nc C	˂LOQ nc C	18.6
*Msa*	˂LOQ cC	˂LOQ cC	˂LOQ nc NC	˂LOQ nc NC	˂LOQ
*Mlu*	˂LOQ cC	˂LOQ cC	˂LOQ nc NC	˂LOQ nc NC	˂LOQ
*Ovi*	˂LOQ cC	˂LOQ cC	˂LOQ nc NC	˂LOQ nc NC	˂LOQ
*Agl*	˂LOQ cC	˂LOQ cC	˂LOQ nc NC	˂LOQ nc NC	˂LOQ
*Aci*	˂LOQ cC	˂LOQ cC	˂LOQ nc NC	˂LOQ nc NC	˂LOQ
AVG*	6.4	4.5	nc	nc	10.8
**Sprouted seeds**					
*Tpr*	15.2 ± 1.3 bB	4.9 ± 0.5 bB	1.0 ± 0.1 bB	3.5 ± 0.4 bB	24.6
*Tme*	71.3 ± 4.5 aB	16.5 ± 1.3 aB	1.6 ± 0.2 aB	7.8 ± 0.7 aB	97.2
*Msa*	3.3 ± 0.3 cB	1.6 ± 0.2 cA	˂LOQ nc NC	˂LOQ nc NC	4.9
*Mlu*	1.8 ± 0.9 cA	1.4 ± 0.2 cA	˂LOQ nc NC	˂LOQ nc NC	3.2
*Ovi*	2.6 ± 1.7 cA	2.3 ± 0.3 cA	˂LOQ nc NC	˂LOQ nc NC	4.9
*Agl*	1.3 ± 0.2 cB	0.8 ± 0.1 cB	˂LOQ nc NC	˂LOQ nc NC	2.1
*Aci*	1.6 ± 1.0 cB	1.1 ± 0.2 cA	˂LOQ nc NC	˂LOQ nc NC	2.7
AVG*	43.3	10.7	1.3	5.7	60.9
AVG**	2.14	1.42	nc	nc	3.56
**Microgreens**					
*Tpr*	89.9 ± 5.3 bA	19.9 ± 1.3 bA	1.4 ± 0.2 bA	1.3 ± 1.0 bA	112.5
*Tme*	117.5 ± 6.3 aA	33.4 ± 2.1 aA	2.7 ± 0.3 aA	17.4 ± 1.4 aA	171.0
*Msa*	4.4 ± 0.4 cA	1.5 ± 0.2 cA	˂LOQ nc NC	˂LOQ nc NC	5.9
*Mlu*	2.2 ± 0.3 cA	1.8 ± 0.3 cA	˂LOQ nc NC	˂LOQ nc NC	4.0
*Ovi*	2.9 ± 0.4 cA	2.1 ± 0.2 cA	˂LOQ nc NC	˂LOQ nc NC	5.0
*Agl*	2.3 ± 0.3 cA	1.5 ± 0.2 cA	˂LOQ nc NC	˂LOQ nc NC	3.8
*Aci*	2.1 ± 0.3 cA	1.2 ± 0.2 cA	˂LOQ nc NC	˂LOQ nc NC	3.3
AVG*	103.7	26.7	2.1	9.4	141.8
AVG**	2.56	1.84	nc	nc	4.4

AVG*-average for *Trifolium* species, AVG**-average for all remaining species, nc, NC-not computed. Data with isoflavone concentration <LOQ (limits of quantification) were not included for calculation of total isoflavone. The different lowercase letters (a, b, c) in the column indicate significant differences (*p* < 0.05) in the respective isoflavone concentrations among the legume accessions within the identical ontogenic stage and the different uppercase letters (A, B, C) in the column indicate significant differences (*p* < 0.05) in the respective isoflavone concentrations among ontogenic stages within the identical legume species. For computation, LOQ values (indicated in Materials and Methods) of formononetin and biochanin A were included for the isoflavone concentrations <LOQ. Abreviations: *Tpr-T. pratense, Tme-T. medium, Msa-M. sativa, Mlu-M. lupulina, Ovi-O. viciifolia, Agl-A. glycyphyllos, Aci-A. cicer.*

**Table 4:** The alternation of the concentrations of coumestrol, triterpene saponins and condensed tannins in the chain raw seeds-sprouted seeds-microgreens of perennial legumes.

Compounds	Sample	*M. sativa*	*M. lupulina*	*O. viciifolia*
		Concentration, mg/100 g ± SD		
Coumestrol	Raw seeds	˂LOQ nc NC	˂LOQ nc NC	˂LOQ
	Sprouted seeds	0.41 ± 0.06 bB	1.12 ± 0.14 aB	˂LOQ
	Microgreens	1.27 ± 0.14 bA	4.56 ± 0.33 aA	˂LOQ
Triterpene saponins	Raw seeds	121 ± 21.1 aC	68.0 ± 15.6 bC	˂LOQ
	Sprouted seeds	286 ± 34.9 aB	305 ± 47.8 aA	˂LOQ
	Microgreens	484 ± 66.0 aA	411 ± 5.27 bA	˂LOQ
Condensed tannins	Raw seeds	˂LOQ	˂LOQ	˂LOQ NC
	Sprouted seeds	˂LOQ	˂LOQ	388 ± 45.5 B
	Microgreens	˂LOQ	˂LOQ	449 ± 51.7 A

The different lowercase letters (a, b, c) indicate significant differences (*p* < 0.05) in the respective metabolite concentrations between *Medicago* species within the identical ontogenic stage and the different uppercase letters (A, B, C) in the column indicate significant differences (*p* < 0.05) in the respective compound concentrations among ontogenic stages within the identical legume accession nc, NC-data with metabolite concentration <LOQ were not included for computation.

**Table 5:** A list of the studied perennial legume species from the sub-family *Faboideae*.

Tribe	Genus	Scientific Name (Abbreviation)	English Name	Cultivar/Wild Ecotype	Country of Origin, Geographic Coordinates
*Trifolieae*	*Trifolium*	*T. pratense* (*Tpr*)	Red clover	‘Sadūnai’	Lithuania, 55°23′49″ N; 23°51′40″ E
*Trifolieae*	*Trifolium*	*T. medium* (*Tme*)	Zigzag clover	Wild ecotype	Lithuania 55°32′42″ N; 25°02′23″ E
*Trifolieae*	*Medicago*	*M. sativa* (*Msa*)	Alfalfa	‘Birutė’	Lithuania, 55°23′49″ N; 23°51′40″ E
*Trifolieae*	*Medicago*	*M. lupulina* (*Mlu*)	Black medick	‘Arka’	Lithuania, 55°23′49″ N; 23°51′40″ E
*Hedysareae*	*Onobrychis*	*O. viciifolia* (*Ovi*)	Sainfoin	‘Meduviai’	Lithuania, 55°23′49″ N; 23°51′40″ E
*Galegeae*	*Astragalus*	*A. glycyphyllos* (*Agl*)	Liquorice milkvetch	Wild ecotype	Lithuania, 55°22′51″ N; 23°50′35″ E
*Galegeae*	*Astragalus*	*A. cicer* (*Aci*)	Cicer milkvetch	Wild ecotype	Latvia, 57°01′45″ N; 21°25′23″ E

## References

[1] Muzquiz M., Varela A., Burbano C., Cuadrado C., Guillamón E., Pedrosa M.M. (2012). Bioactive compounds in legumes: Pronutritive and antinutritive actions. Implications for nutrition and health. Phytochem. Rev..

[2] Shahidi F. (2004). Functional foods: Their role in health promotion and disease prevention. J. Food Sci..

[3] International Legume Database & Information Service World Database of Legumes. http://www.ildis.org/.

[4] Bouchenak M., Lamri-Senhadji M. (2013). Nutritional quality of legumes, and their role in cardiometabolic risk prevention: A review. J. Med. Food..

[5] Weide A., Riehl S., Zeidi M., Conard N.J. (2017). Reconstructing subsistence practices: Taphonomic constraints and the interpretation of wild plant remains at aceramic Neolithic Chogha Golan, Iran. Veg. Hist. Archaeobot..

[6] Butler A. (1995). The small-seeded legumes: An enigmatic prehistoric resource. Acta Palaeobot..

[7] Dini C., García M.A., Vina S.Z. (2012). Non-traditional flours: Frontiers between ancestral heritage and innovation. Food Funct..

[8] Bora K.S., Sharma A. (2011). Phytochemical and pharmacological potential of *Medicago sativa*: A review. Pharm. Biol..

[9] Łuczaj Ł. (2012). Ethnobotanical review of wild edible plants of Slovakia. Acta Soc. Bot. Pol..

[10] Ionkova I. (2008). Anticancer compounds from in vitro cultures of rare medicinal plants. Pharmacogn. Rev..

[11] Adams F. (1847). The Seven Books of Paulus Aegineta. Translated from Greek. With a Commentary Embracing a Complete View of the Knowledge Possessed by the Greeks, Romans, and Arabians on all Subjects Connected with Medicine and Surgery.

[12] Malisch C.S., Luscher A., Baert N., Engstrom M.T., Studer B., Fryganas C., Suter D., Mueller-Harvey I., Salminen J.P. (2015). Large variability of proanthocyanidin content and composition in sainfoin (*Onobrychis viciifolia*). J. Agric. Food Chem..

[13] Saviranta N.M.M., Anttonen M.J., von Wright A., Karjalainen R.O. (2008). Red clover (*Trifolium pratense* L.) isoflavones: Determination of concentrations by plant stage, flower colour, plant part and cultivar. J. Sci. Food Agric..

[14] Butkutė B., Dagilytė A., Benetis R., Padarauskas A., Cesevičienė J., Olšauskaitė V., Lemežienė N. (2018). Mineral and phytochemical profiles and antioxidant activity of herbal material from two temperate *Astragalus* species. BioMed Res. Int..

[15] Butkutė B., Lemežienė N., Padarauskas A., Norkevičienė E., Taujenis L., Brazauskas G., Statkevičiūtė G., Jonavičienė K. (2018). Chemical composition of zigzag clover (*Trifolium medium* L.). Breeding Grasses and Protein Crops in the Era of Genomics.

[16] Butkutė B., Padarauskas A., Cesevičienė J., Taujenis L., Norkevičienė E. (2018). Phytochemical composition of temperate perennial legumes. Crop Pasture Sci..

[17] Butkutė B., Benetis R., Padarauskas A., Cesevičienė J., Dagilytė A., Taujenis L., Rodovičius H., Lemežienė N. (2017). Young herbaceous legumes—A natural reserve of bioactive compounds and antioxidants for healthy food and supplements. J. Appl. Bot. Food Qual..

[18] Butkutė B., Padarauskas A., Cesevičienė J., Pavilonis A., Taujenis L., Lemežienė N. (2017). Perennial legumes as a source of ingredients for healthy food: Proximate, mineral and phytoestrogen composition and antibacterial activity. J. Food Sci. Technol..

[19] Prati S., Baravelli V., Fabbri D., Schwarzinger C., Brandolini V., Maietti A., Tedeschi P., Benvenuti S., Macchia M., Marotti I. (2007). Composition and content of seed flavonoids in forage and grain legume crops. J. Sep. Sci..

[20] Cos P., De Bruyne T., Hermans N., Apers S., Vanden Berghe D., Vlietinck A.J. (2004). Proanthocyanidins in health care: Current and new trends. Curr. Med. Chem..

[21] Seguin P., Zheng W. (2006). Phytoestrogen content of alfalfa cultivars grown in eastern Canada. J. Sci. Food Agric..

[22] Boe A., Bortnem R., Johnson P.J. (2016). Changes in weight and germinability of black medic seed over a growing season, with a new seed predator. Proc. S. D. Acad. Sci..

[23] Agelet A., Valles J. (2003). Studies on pharmaceutical ethnobotany in the region of Pallars (Pyrenees, Catalonia, Iberian Peninsula). Part II. New or very rare uses of previously known medicinal plants. J. Ethnopharmacol..

[24] Aguilera Y., Díaz M.F., Jiménez T., Benítez V., Herrera T., Cuadrado C., Martín-Pedrosa M., Martín-Cabrejas M.A. (2013). Changes in nonnutritional factors and antioxidant activity during germination of nonconventional legumes. J. Agric. Food Chem..

[25] Cevallos-Casals B.A., Cisneros-Zevallos L. (2010). Impact of germination on phenolic content and antioxidant activity of 13 edible seed species. Food Chem..

[26] Plaza L., de Ancos B., Cano M.P. (2003). Nutritional and health-related compounds in sprouts and seeds of soybean (*Glycine max*), wheat (*Triticum aestivum* L.) and alfalfa (*Medicago sativa*) treated by a new drying method. Eur. Food Res. Technol..

[27] Danilčenko H., Dabkevičius Z., Jarienė E., Tarasevičienė Ž., Televičiūtė D., Tamošiūnas A., Jeznach M. (2017). The effect of stinging nettle and field horsetail extracts on the synthesis of biologically active compounds in germinated leguminous and quinoa seed. Zemdirbyste.

[28] Ebert A.W., Wu T.H., Yang R.Y., Hughes J.d., Kasemsap P., Dasgupta S., Dutta O.P., Ketsa S., Chaikiattiyos S., Linwattana G., Kosiyachinda S., Chantrasmi V. (2014). Amaranth sprouts and microgreens–a homestead vegetable production option to enhance food and nutrition security in the rural-urban continuum. Proceedings of the Regional Symposium on Sustaining Small-Scale Vegetable Production and Marketing Systems for Food and Nutrition Security (SEAVEG2014).

[29] Márton M., Mándoki Z., Csapo J. (2010). Evaluation of biological value of sprouts I. Fat content, fatty acid composition. Acta Univ. Sapientiae Aliment..

[30] Oleszek W.A. (1998). Composition and quantitation of saponins in alfalfa (*Medicago sativa* L.) seedlings. J. Agric. Food Chem..

[31] Budryn G., Gałązka-Czarnecka I., Brzozowska E., Grzelczyk J., Mostowski R., Żyżelewicz D., Cerón-Carrasco J.P., Pérez-Sánchez H. (2018). Evaluation of estrogenic activity of red clover (*Trifolium pratense* L.) sprouts cultivated under different conditions by content of isoflavones, calorimetric study and molecular modelling. Food Chem..

[32] Baldinger L., Hagmüller W., Minihuber U., Matzner M., Zollitsch W. (2016). Sainfoin seeds in organic diets for weaned piglets—Utilizing the protein-rich grains of a long-known forage legume. Renew. Agric. Food.

[33] Ebert A.W., Chang C.H., Yan M.R., Yang R.Y. (2017). Nutritional composition of mungbean and soybean sprouts compared to their adult growth stage. Food Chem..

[34] Dueñas M., Sarmento T., Aguilera Y., Benitez V., Mollá E., Esteban R.M., Martín-Cabrejas M.A. (2016). Impact of cooking and germination on phenolic composition and dietary fibre fractions in dark beans (*Phaseolus vulgaris* L.) and lentils (*Lens culinaris* L.). LWT-Food Sci. Technol..

[35] Hedley C.L., Hedley C.L. (2000). Grain legume carbohydrates. Carbohydrates in Grain Legume Seeds: Improving Nutritional Quality and Agronomic Characteristics.

[36] Masood T., Shah H.U., Zeb A. (2014). Effect of sprouting time on proximate composition and ascorbic acid level of mung bean (*Vigna radiate* L.) and chickpea (*Cicer Arietinum* L.) seeds. J. Anim. Plant Sci..

[37] Maneemegalai S., Nandakumar S. (2011). Biochemical studies on the germinated seeds of *Vigna radiata* (L.) R. Wilczek, *Vigna mungo* (L.) Hepper and *Pennisetum typhoides* (Burm f.) Stapf and CE Hubb. Int. J. Agric. Res..

[38] Machado A.L.D.L., Barcelos M.D.F.P., Teixeira A.H.R., Nogueira D.A. (2009). Evaluation of chemical compounds in Fabaceae sprouts for the human consumption. Cienc. Agrotec..

[39] Khalil A.W., Zeb A., Mahmood F., Tariq S., Khattak A.B., Shah H. (2007). Comparison of sprout quality characteristics of desi and kabuli type chickpea cultivars (*Cicer arietinum* L.). LWT-Food Sci. Technol..

[40] Bau H.M., Villaume C., Nicolas J.P., Méjean L. (1997). Effect of germination on chemical composition, biochemical constituents and antinutritional factors of soya bean (*Glycine max*) seeds. J. Sci. Food Agric..

[41] Devi C.B., Kushwaha A., Kumar A. (2015). Sprouting characteristics and associated changes in nutritional composition of cowpea (Vigna unguiculata). J. Food Sci. Technol..

[42] White P.J., Broadley M.R. (2009). Biofortification of crops with seven mineral elements often lacking in human diets–iron, zinc, copper, calcium, magnesium, selenium and iodine. New Phytol..

[43] Dwyer J.T., Picciano M.F., Betz J.M., Fisher K.D., Saldanha L.G., Yetley E.A., Coates P.M., Radimer K., Bindewald B., Sharpless K.E. (2006). Progress in development of an integrated dietary supplement ingredient database at the NIH Office of Dietary Supplements. J. Food Compost. Anal..

[44] Iqbal A., Khalil I.A., Ateeq N., Khan M.S. (2006). Nutritional quality of important food legumes. Food Chem..

[45] Sangronis E., Machado C.J. (2007). Influence of germination on the nutritional quality of Phaseolus vulgaris and Cajanus cajan. LWT-Food Sci. Technol..

[46] Özcan M.M., Al Juhaimi F. (2014). Effect of sprouting and roasting processes on some physico-chemical properties and mineral contents of soybean seed and oils. Food Chem..

[47] Adatorwovor R., Roggenkamp K., Anderson J. (2015). Intakes of calcium and phosphorus and calculated calcium-to-phosphorus ratios of older adults: NHANES 2005–2006 data. Nutrients.

[48] Kemi V.E., Kärkkäinen M.U., Rita H.J., Laaksonen M.M., Outila T.A., Lamberg-Allardt C.J. (2010). Low calcium: Phosphorus ratio in habitual diets affects serum parathyroid hormone concentration and calcium metabolism in healthy women with adequate calcium intake. Br. J. Nutr..

[49] McClure S.T., Chang A.R., Selvin E., Rebholz C.M., Appel L.J. (2017). Dietary sources of phosphorus among adults in the United States: Results from NHANES 2001–2014. Nutrients.

[50] Dave S., Yadav B.K., Tarafdar J.C. (2008). Phytate phosphorus and mineral changes during soaking, boiling and germination of legumes and pearl millet. J. Food Sci. Technol..

[51] Bains K., Uppal V., Kaur H. (2014). Optimization of germination time and heat treatments for enhanced availability of minerals from leguminous sprouts. J. Food Sci. Technol..

[52] Karadima V., Kraniotou C., Bellos G., Tsangaris G.T. (2016). Drug-micronutrient interactions: Food for thought and thought for action. EPMA J..

[53] Gregory P.J., Wahbi A., Adu-Gyamfi J., Heiling M., Gruber R., Joy E.J., Broadley M.R. (2017). Approaches to reduce zinc and iron deficits in food systems. Glob. Food Secur..

[54] La Frano M.R., De Moura F.F., Boy E., Lönnerdal B., Burri B.J. (2014). Bioavailability of iron, zinc, and provitamin A carotenoids in biofortified staple crops. Nutr. Rev..

[55] Hunt J.R. (2003). Bioavailability of iron, zinc, and other trace minerals from vegetarian diets. Am. J. Clin. Nutr..

[56] Luo Y.W., Xie W.H., Jin X.X., Wang Q., He Y.J. (2014). Effects of germination on iron, zinc, calcium, manganese, and copper availability from cereals and legumes. CYTA-J. Food..

[57] Urbano G., López-Jurado M., Aranda C., Vilchez A., Cabrera L., Porres J.M., Aranda P. (2006). Evaluation of zinc and magnesium bioavailability from pea (*Pisum sativum*, L.) sprouts. Effect of illumination and different germination periods. Int. J. Food Sci. Technol..

[58] Forslund L.C., Andersson H.C. (2017). Phytoestrogens in Foods on the Nordic Market: A Literature Review on Occurrence and Levels. https://www.diva-portal.org/smash/get/diva2:1137191/fulltext01.pdf.

[59] Mattioli S., Dal Bosco A., Martino M., Ruggeri S., Marconi O., Sileoni V., Falcinelli B., Castellini C., Benincasa P. (2016). Alfalfa and flax sprouts supplementation enriches the content of bioactive compounds and lowers the cholesterol in hen egg. J. Funct. Foods.

[60] Wu Z., Song L., Feng S., Liu Y., He G., Yioe Y., Liu S.Q., Huang D. (2012). Germination dramatically increases isoflavonoid content and diversity in chickpea (*Cicer arietinum* L.) seeds. J. Agric. Food Chem..

[61] López A., El-Naggar T., Dueñas M., Ortega T., Estrella I., Hernández T., Gómez-Serranillos M.P., Palomino O.M., Carretero M.E. (2013). Effect of cooking and germination on phenolic composition and biological properties of dark beans (*Phaseolus vulgaris* L.). Food Chem..

[62] Lee S.J., Ahn J.K., Khanh T.D., Chun S.C., Kim S.L., Ro H.M., Song H.K., Chung I.M. (2007). Comparison of isoflavone concentrations in soybean (*Glycine max* (L.) Merrill) sprouts grown under two different light conditions. J. Agric. Food Chem..

[63] Lin P.Y., Lai H.M. (2006). Bioactive compounds in legumes and their germinated products. J. Agric. Food Chem..

[64] Jeon H.Y., Seo D.B., Shin H.J., Lee S.J. (2012). Effect of Aspergillus oryzae-challenged germination on soybean isoflavone content and antioxidant activity. J. Agric. Food Chem..

[65] Shimoyamada M., Okubo K. (1991). Variation in saponin contents in germinating soybean seeds and effect of light irradiation. Agric. Biol. Chem..

[66] Singh B., Singh J.P., Singh N., Kaur A. (2017). Saponins in pulses and their health promoting activities: A review. Food Chem..

[67] Pecetti L., Tava A., Romani M., De Benedetto M.G., Corsi P. (2006). Variety and environment effects on the dynamics of saponins in lucerne (*Medicago sativa* L.). Eur. J. Agron..

[68] Troszyńska A., Wołejszo A., Narolewska O. (2006). Effect of germination time on the content of phenolic compounds and sensory quality of mung bean (*Vigna radiata* L.) sprouts. Pol. J. Food Nutr. Sci..

[69] Kalpanadevi V., Mohan V.R. (2013). Effect of processing on antinutrients and in vitro protein digestibility of the underutilized legume, *Vigna unguiculata* (L.) Walp subsp. unguiculata. LWT-Food Sci. Technol..

[70] Świeca M., Gawlik-Dziki U., Kowalczyk D., Złotek U. (2012). Impact of germination time and type of illumination on the antioxidant compounds and antioxidant capacity of *Lens culinaris* sprouts. Sci. Hortic..

[71] Klopsch R., Baldermann S., Voss A., Rohn S., Schreiner M., Neugart S. (2018). Bread enriched with legume microgreens and leaves–ontogenetic and baking-driven changes in the profile of secondary plant metabolites. Front. Chem..

[72] Cornara L., Xiao J., Burlando B. (2016). Therapeutic potential of temperate forage legumes: A review. Crit. Rev. Food Sci. Nutr..

[73] Owusu-Apenten R. (2004). Introduction to Food Chemistry.

[74] Leśniewicz A., Jaworska K., Żyrnicki W. (2006). Macro-and micro-nutrients and their bioavailability in polish herbal medicaments. Food Chem..

[75] Pecetti L., Biazzi E., Tava A. (2010). Variation in saponin content during the growing season of spotted medic (*Medicago arabica* (L.) Huds.). J. Agric. Food Chem..

[76] Taujenis L., Padarauskas A., Cesevičienė J., Lemežienė N., Butkutė B. (2016). Determination of coumestrol in lucerne by ultra-high pressure liquid chromatography-mass spectrometry. Chemija.

